# The Aachen model study course in medicine – development and implementation. Fifteen years of a reformed medical curriculum at RWTH Aachen University

**DOI:** 10.3205/zma001268

**Published:** 2019-10-15

**Authors:** Melanie Simon, Annika Martens, Sonja Finsterer, Sandra Sudmann, Johann Arias

**Affiliations:** 1 RWTH Aachen University, Faculty of Medicine, Office of the Dean of Studies, Aachen, Germany

**Keywords:** curriculum development, reformed curricula, problem-based learning

## Abstract

**Objective: **The Aachen model study course in medicine was developed in response to a negative appraisal of the Faculty of Medicine of the RWTH Aachen University by the Science Council in 2000. The aim is to create graduates who are capable of further training and can work in evidence-based and patient-centered health care while incorporating scientific findings.

**Methodology:** In 2003 the medical degree was fully switched over to the model study course format. This means an annual cohort size of about 280 students. These go through a modularized and integrated curriculum, which is designed as a learning spiral. This requires a special interdisciplinary collaboration of teachers and curriculum planners. In addition to the modules, longitudinal elements such as workplace-based examinations, communication or practical skills are embedded in the curriculum.

**Results: **The state exam results of the Aachen graduates have already improved significantly even for the first cohort and the university has been able to maintain an almost uninterrupted high level from 2008 to 2018. The students satisfaction with the model course is not only evident in the student course assessment and qualitative group discussions but also in various national rankings.

**Conclusion: **The complete redesign of the course starting from the first semester onwards posed major challenges for all those involved in the faculty. The implementation of a completely reformed curriculum, such as the model study course, can only succeed through constructive cooperation of the various stakeholders at a faculty. The reorientation was able to address the major flaws of the 2002 report by the Science Council, student dissatisfaction and the poor performance in the nationwide state exams.

## Zusammenfassung

**Zielsetzung:** Der Aachener Modellstudiengang Medizin wurde als Reaktion der Medizinischen Fakultät der RWTH Aachen auf eine negative Beurteilung durch den Wissenschaftsrat im Jahr 2000 entwickelt. Ziel sind weiterbildungsfähige Absolventen und Absolventinnen, die in der Lage sind, evidenzbasierte und patientenzentrierte Krankenversorgung zu betreiben und dabei wissenschaftliche Erkenntnisse einbeziehen.

**Methodik: **Das Studium der Humanmedizin wurde im Jahr 2003 insgesamt auf den Modellstudiengang umgestellt. Dies bedeutet eine jährliche Kohortengröße von ca. 280 Studierenden. Diese durchlaufen ein modularisiertes und integriertes Curriculum, welches sich im Sinne einer Lernspirale gestaltet. Dies erfordert eine besondere interdisziplinäre Zusammenarbeit von Lehrenden und Curriculumsplanern. Neben den Modulen sind longitudinale Elemente wie das arbeitsplatzbasierte Prüfen, die Kommunikation oder praktische Fertigkeiten in das Curriculum eingebettet.

**Ergebnisse: **Die Staatsexamensergebnisse der Aachener Absolventen und Absolventinnen haben sich schon ab dem ersten Jahrgang deutlich verbessert und konnten ein hohes Niveau von 2008 bis 2018 fast ununterbrochen halten. Die Studierenden drücken ihre Zufriedenheit mit dem Modellstudiengang nicht nur in der studentischen Lehrveranstaltungsbewertung und in qualitativen Gruppengesprächen, sondern auch in diversen nationalen Rankings aus.

**Schlussfolgerung:** Die völlige Neukonzeptionierung des Studiengangs ab dem ersten Semester aufbauend stellte alle Beteiligten an der Fakultät vor große Herausforderungen. Die Umsetzung eines komplett reformierten Curriculums, wie sie die Einführung des MSG darstellte, gelingt nur durch eine konstruktive Zusammenarbeit verschiedener Akteure einer Fakultät. Die neue Ausrichtung konnte die Hauptmängel des WR Berichtes aus dem Jahre 2002, die Unzufriedenheit der Studierenden und das schlechte Abschneiden im Examen im bundesweiten Vergleich, beheben.

## 1. Occasion, need

The Faculty of Medicine of RWTH Aachen University was extensively evaluated in 1999 on the basis of a report written by the faculty and an inspection by the Science Council. This evaluation culminated in a statement by the Science Council in December 2000 on the future development of the Faculty of Medicine. In addition to assessing and evaluating the research orientation of the faculty, the medical education of students was also assessed in this context. Overall, the Science Council questioned the academic standards of the faculty and judged that it would not be competitive as a university medical school. This was the beginning of a major reorientation for the faculty. With regard to the teaching and study of medicine, the Scientific Council criticized in particular the below-average exam results of the graduates in the Intermediate Examination in Medical Studies and State Examinations and the poor organization of teaching by the faculty [1].

Both this report by the Science Council, together with the model study course clause of the Medical Licensure Act of 200, laid the foundation for the faculty’s decision to switch the entire medical degree in Aachen to the model study course [https://www.gesetze-im-internet.de/_appro_2002/BJNR240500002.html]. The Aachen model study course in medicine (MSCM) started in the winter semester 2003/2004 with 280 students. The standard degree course of study was successively discontinued [2].

The integrated curriculum of the MSCM was developed under the direction of the former Dean of Studies, Prof. Rolf Rossaint, in cooperation with the medical student council. A key role in its conception was played by Prof. Peter Kaufmann from the then Anatomical Institute 2, who oversaw the MSCM until his death in 2010. The anatomical institutes are still significantly involved in the implementation and further development of the MSCM. Their teaching staff oversee several interdisciplinary modules.

## 2. Aims

The first training goal of the MSCM is to produce academically qualified doctors capable of further training who have acquired cognitive knowledge, practical skills, scientific and communicative skills and a work attitude that will enable lifelong learning in all of these areas. The MSCM students are to be scientifically trained and familiarized with the process of biological and medical problem solving. Their training is designed in a way that the students do not acquire subject-specific factual knowledge but understand organ systems in their construction, their function and their pathogenetic principles [3]. 

The second training goal is the acquisition of the ability to learn and think in an integrative cross-disciplinary way and to reflect their own knowledge and experience in a self-critical way. Through interdisciplinary teaching of content, students learn at an early stage to look at biological systems and matters relating to them from different points of view. This should help them in their future activity to incorporate differential diagnostic and therapeutic considerations into their medical practice. These goals are also anchored in the profile of the MSCM, in which the overarching learning objectives have been formulated [4], [5], [6].

The development of the MSCM specific learning objective catalog has also increased the transparency of learning objectives and provided a sound basis for discussions on teaching and studying. The learning objective catalog was adopted at a closed session of the model study course in 2009 and developed in the following two years by the Institute for Medical Information Technology in the form of a Wiki and filled with content by the specialist representatives of the hospitals and institutes. The process was moderated by a working group staffed with representatives from both the clinical and pre-clinical phase of studies. The Wiki format allows the faculty to continue developing the catalog to this day [7].

## 3. Teaching methods, teaching formats

### 3.1. Phases of study

**Figure 1:**
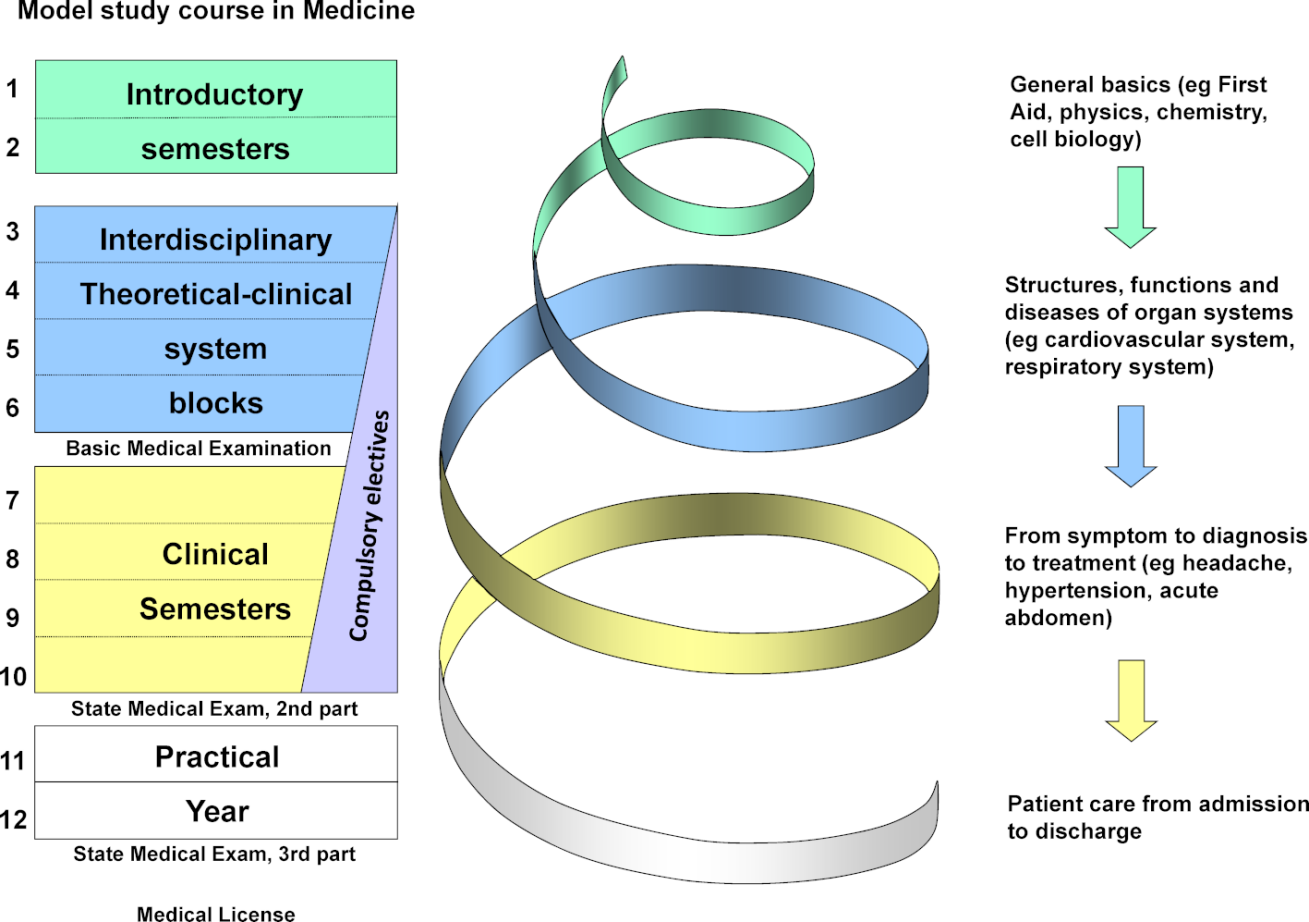
Learning spiral in the model study course in medicine

Medical studies in the MSCM is divided into four phases of study (see figure 1 (Fig. 1)). 

#### 3.1.1. 1. Phase of studies – Homogenization

Medical studies begins with a three-week introductory block. This provides a first insight into the professional field and teaches medical hygiene and emergency procedures. In further courses, the disparate prior scientific knowledge of the students is brought to a comparable level. In this context, the course in cell biology is a fundamental component of the first academic year. An interdisciplinary preparatory course of the organ systems (IPO) provides basic knowledge about the structures and functions of the human body. 

#### 3.1.2. 2. Phase of studies – Interdisciplinary modules

The second phase of studies consists of interdisciplinary theoretical-clinical modules. In this phase, clinical content is taught for the first time from the third semester on. Modules deal with organ systems of the body in a modular manner in lectures, seminars, seminars for problem-based learning (Pbl) and internships (see figure 2 (Fig. 2)). 

The learning spiral is traversed here for the second time starting from the organ. In addition to the interdisciplinary modules, there are cross-sectional subjects that deal with cross-organ content in lectures, exercises and internships. In the second phase of studies the medical examination course (EC) will be offered parallel to the modules from the third semester onwards. Various examination techniques are first practiced by students amongst themselves or using standardized patients. In the following, patients are examined by students in groups of three under medical supervision, communication techniques are trained and special examination techniques are taught. The skills learned are formally reviewed within the EC using a workplace-based assessment format, the MINI Clinical Evaluation Exercise (Mini-CEX) [6].

Passing the Basic Medical Examination (ÄBP), the M1 equivalent of the MSCM, is a prerequisite for admission to the third phase of studies.

#### 3.1.3 3. Phase of studies – Clinical semesters

In this phase of studies patients and their illnesses are placed at the center of teaching. Sicknesses are no longer viewed from the organ’s point of view but are considered according to the patients and their symptoms. An important part of this phase of studies are the clinical block internships, which take place for half the cohort in the 8^th^ and 9^th^ semesters. The other half of the cohort passes through the so-called “Free Elective Semester”, which offers time for medical internships, the preparation of a doctoral thesis or stays abroad. The 10^th^ semester is dedicated to economic, health, social and ethical aspects of medicine and concludes with the Course of Clinical Competence (CCC). For the students this phase of studies will conclude with the second part of the State Medical Exam (M2).

#### 3.1.4 4. Phase of studies – Practical Year 

The Practical Year (PY) is divided into three 4-month rotations in the MSCM too and these are completed in internal medicine, surgery and a subject of the student’s choice. The PY begins with a PY preparatory course in which the practical skills of the PY students are refreshed at the start of the Practical Year. The PY can be undertaken not only at the Aachen University Hospital but also at academic teaching hospitals. If General Practice is chosen as a 4-month rotation, this is completed in academic teaching surgeries [8].

#### 3.2. Scientific competences in medical studies

**Figure 3:**
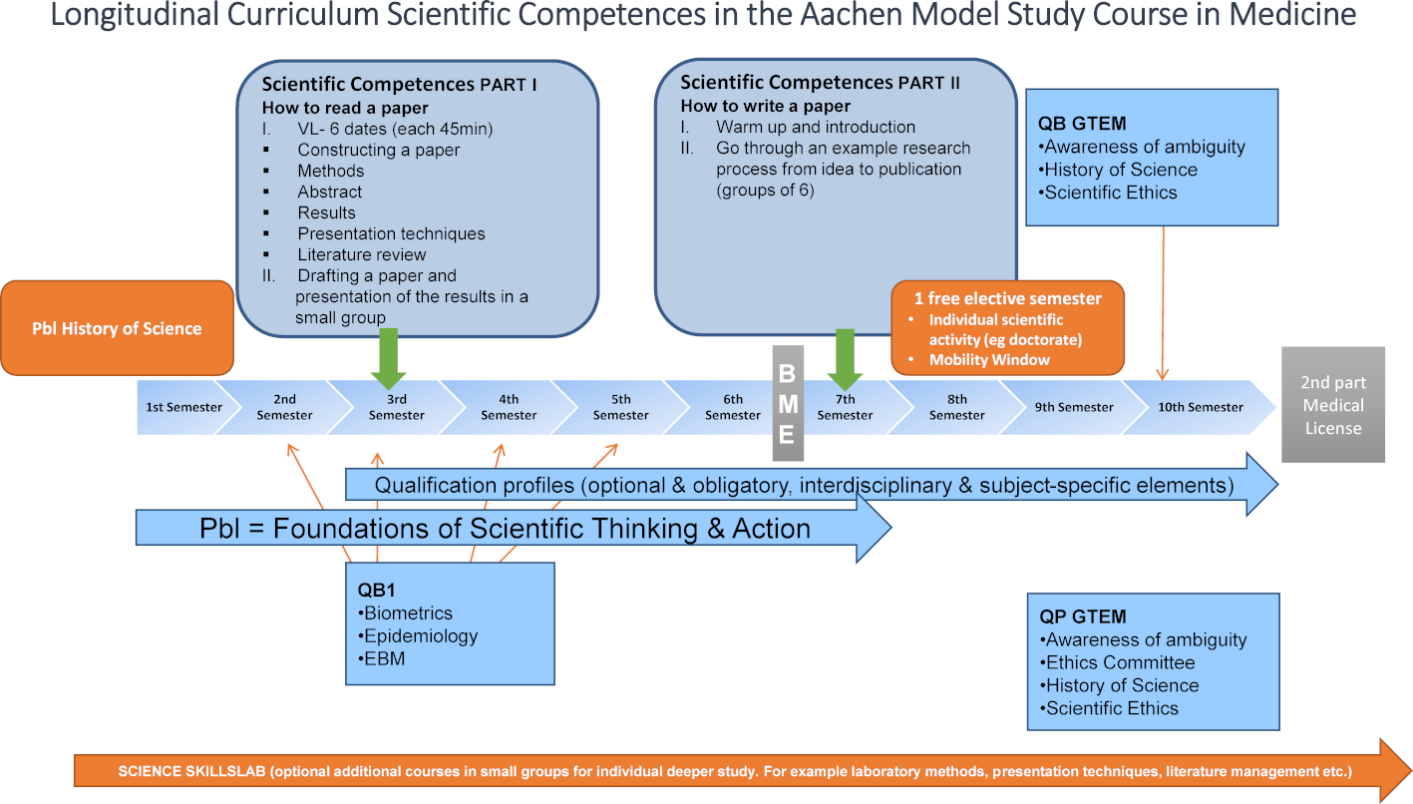
Longitudinal scientific curriculum

Right from the start of medical studies the MSCM pursues the overarching goal of empowering students to think and act scientifically. Skills such as literature research, hypothesis generation, ambiguity awareness or the creation of scientific manuscripts are taught in an interdisciplinary way. From the 1^st^ to the 10^th^ semester, there are compulsory and optional courses for students dealing with a wide variety of aspects of scientific work. By choosing individual qualification profiles, the MSCM lays the foundation for future scientific activity or later specialization in continuing education. Ten percent of the timetable is reserved for these compulsory electives. By focusing compulsory electives on a certain subject area, acquisition of an individual qualification profile is possible. Further elements of this longitudinal curriculum are the Pbl, “How to read and how to write a paper” as well as the science skills lab (see figure 3 (Fig. 3)).

#### 3.3. Problem-based learning (Pbl) 

Problem-based learning (Pbl) is implemented in the curriculum of the MSCM as a longitudinal teaching format from the 1^st^ to the 7^th^ semester. The problem-based and interdisciplinary approach to problems supports the teaching profile of the MSCM and its modularized structure. Using a structured approach, the students practice how to tackle unsolved problems in disease-typical patient cases in a team. In addition, this trains skills that are also essential for scientific work (eg hypothesis generation, literature research) [9]. During their studies, MSCM students complete a total of 14 Pbl cases, each with two dates, in groups of ten students. 

#### 3.4. The AIXTRA skills lab

The AIXTRA training center has established a comprehensive program that uses standardized patients, digital and human, to teach taking a patient’s history and examination techniques; and trains practical and communicative skills with video feedback. Both compulsory courses within the system block framework or other events from the curriculum are offered here as well as optional courses and periods for free practice.

#### 3.5. Teaching and learning culture

The modification of the curriculum into a Z-curriculum, in which pre-clinical and clinical content is integrated, brings special changes to the teaching and learning culture at the teaching, collegial and organizational level [10], [11]. Planning and implementation of teaching requires intensive communication between all participants, including the students. Teaching methods such as Pbl promote the students’ own responsibility for their own learning process [1], [12], [13]. New blended learning concepts give students more choice about the time and place of learning, which has a positive impact on motivation [14]. The desire for innovation and continuous development has also driven the degree of professionalization on the part of teachers through qualifications in medical education. 

Additional emphasis on autonomous learning is provided by the documentation and feedback elements of the student portfolio and the HIP tool (How I Perform).

## 4. Examination system

The examination concept of the MSCM is based on summative and formative examination formats. Early practical teaching requires an early review of practical and communicative skills in the curriculum, alongside exam formats that tend to examine cognitive content [15], [12]. The following examination formats are used in the three phases of studies:

- cognitive: Multiple choice exams, structured oral exams (summative), as well as the Progress Test in Medicine (PTM) (formative)
- psychomotor: Objective Structured Practical Examination (OSPE) (summative), as well as work-place based MiniCEX (formative)

Graded papers and case presentations round off the examination spectrum and promote self-organizational, rhetorical and presentation skills.

### 4.1. Basic Medical Examination (BME)

The Basic Medical Examination (BME) is the MSCM equivalent to the Intermediate Examination in Medical Studies (M1). In accordance with §41 of the Medical Licensure Act, this tests the acquisition of knowledge and skills required for the first section of the State Medical Exam. In addition, clinical content and practical skills are also already tested at this stage because of the MSCM’s interdisciplinary approach. The BME consists of a combined oral and practical exam in the form of an OSPE and a multiple choice exam. 

In order to ensure adequate quality of this university exam, guidelines have been developed which take into account the quality requirements for objectivity, reliability and validity, in accordance with international standards.

**Figure 4:**
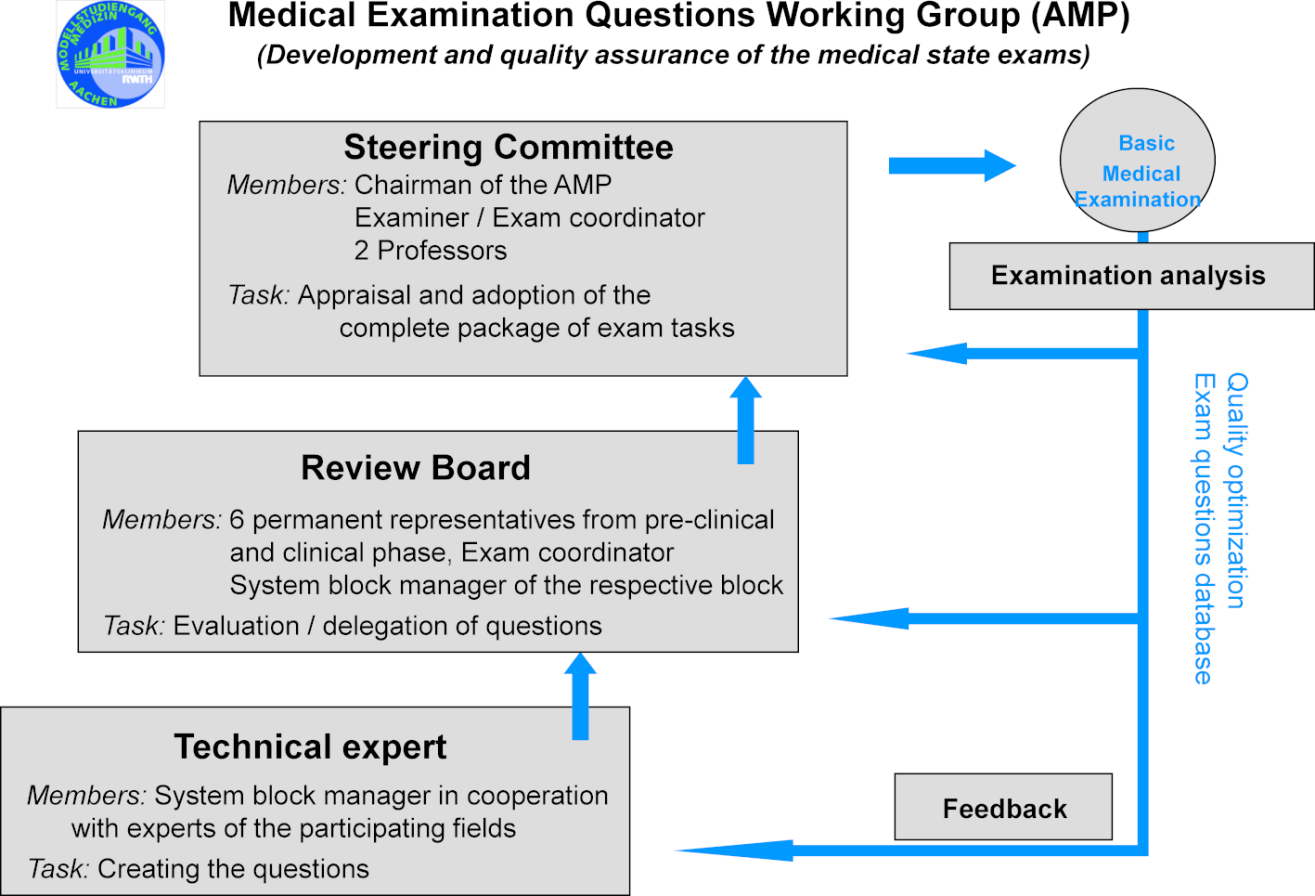
Makeup of the committees involved in problem creation for the basic medical examination

The preparation of the exam assignments goes through a threefold review process, in which the exam contents are checked against the learning objective catalog, the wording of the questions and the horizon of expectation is specified; and the scientific and clinical relevance is reflected. Finally, the approximately 100 exam questions of the oral and practical part and the 120 MC questions are approved by the review panel of the examination board. For quality assurance, there is a re-evaluation (item analysis) documented in writing. Corrections to results and suggestions for improvement are prepared for future examination tasks on the basis of content-related criteria and statistical test evaluation results in this re-evaluation (see figure 4 (Fig. 4)).

#### 4.2. Implementation of a longitudinal concept “MiniCEX”

In 2013, students in the medical student council demanded that more practical exams be integrated into the curriculum and that students receive more feedback about their own skills. The MSCM has therefore initiated a project to implement a longitudinal exam format from workplace-based testing, the Mini Clinical Evaluation Exercise (MiniCEX). In MiniCEX, students from the 3rd to the 10th semester are repeatedly observed clinical situations with patients appropriate to their level of training, as part of the clinical examination course and block internships, and receive oral feedback immediately afterwards. This is documented in the form of a standardized questionnaire. This is an exam format which is purely formative [6].

#### 4.3. Progress Test in Medicine

The Progress Test in Medicine (PTM) is designed as a formative knowledge check and each test consists of 200 MC questions at graduate level. The main use of the PTM is longitudinal feedback for students and comparison with other faculties. Feedback is provided through an online feedback tool called HIP (“How I Perform”). The resulting report allows the students to check their own knowledge gain. According to the examination regulations the PTM is an obligatory offering for all students. Participation in the test is a prerequisite for admission to the next phase of studies [16].

## 5. Evaluation, results

### 5.1. Group and tutor discussions

At the end of each semester, the cohort coordinators hold group and individual discussions with the students. They receive feedback on the general progress of studies and examinations in these discussions. The coordinators are tasked with conveying the results of the discussions constructively to the lecturers and inform students about support offers and similar assistance. Therefore these discussions contribute to a continuous improvement in the organization of the course of studies and the quality of teaching and supplement the compulsory course evaluation by the students.

#### 5.2. Evaluation by the students

The student course evaluation has been carried out since the winter semester 2004/05. The results of the course evaluation are submitted to the study commission and the faculty council and discussed by the student council. 

#### 5.3. Overall findings

Since the beginning of the MSCM, the Faculty of Medicine has been continuously pursuing evidence-based development and improvement of the curriculum. Criticism from the Science Council report of 2000 was successfully addressed. The performance of MSCM graduates today is usually far above the national average [17]. Student satisfaction with the MSCM and its organizational concept is reflected in the various rankings and the lectures. A cross-sectional study also showed that fulfilled expectations are linked to a higher degree of identification with the model study course [18].

The next milestones for the faculty are a mapping of their own learning objective catalog to the National Competence-Based Catalog of Learning Objectives (NKLM) [http://www.nklm.de], [19] as well as the implementation of the Masterplan 2020 [20]. 

**Figure 5:**
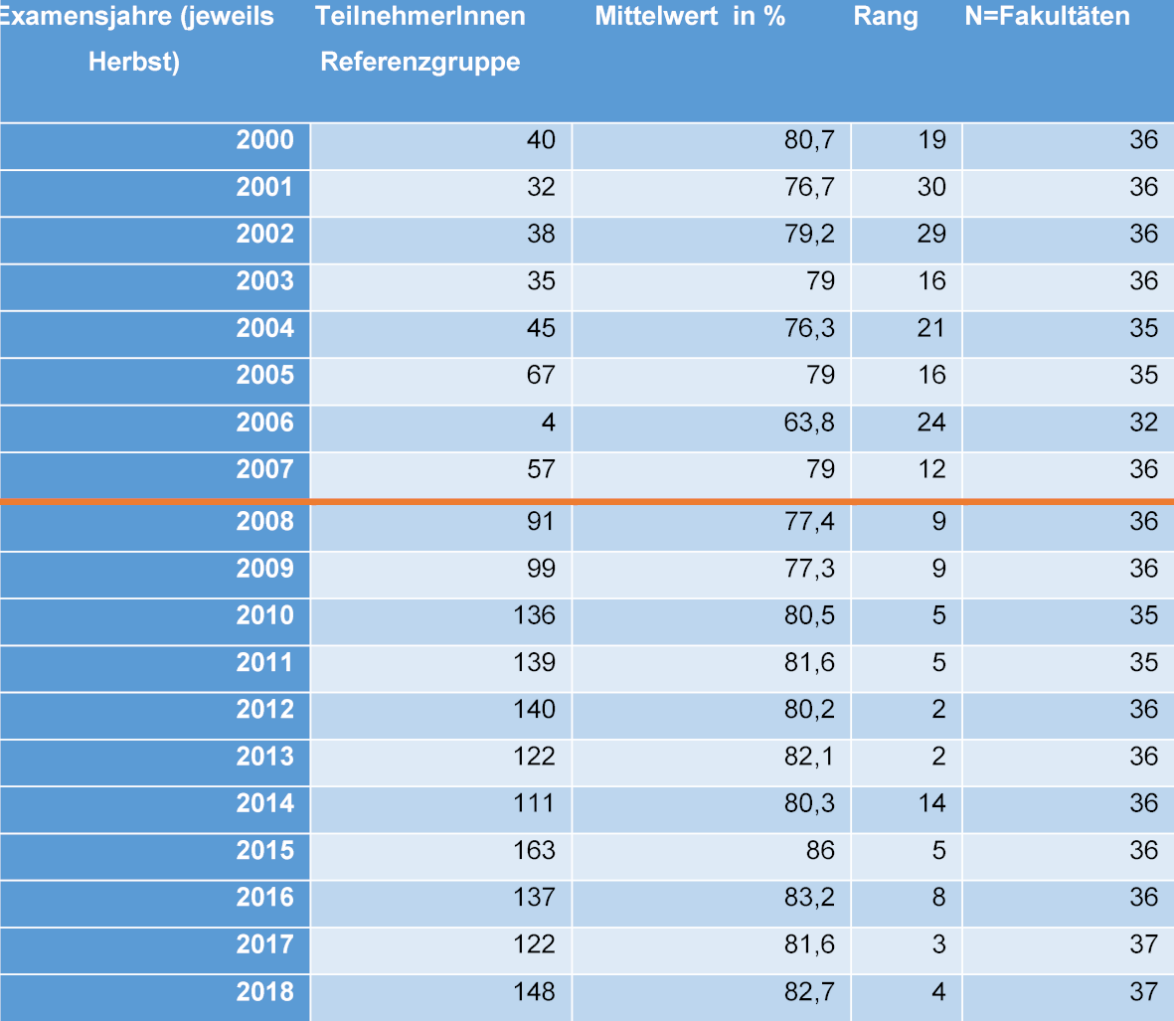
Results of the 2^nd^ part of the state medical exam, Aachen 2000-2018

In October 2018, the Medical Faculty was re-evaluated by the Science Council. The resulting report will show how the Faculty has improved through the introduction of the model study course. Criticisms by the Science Council will feed into the development work in the coming years (see figure 5 (Fig. 5)).

## 6. Miscellaneous

### 6.1. Involved parties

#### 6.1.1. Office of the Dean and Office of the Dean of Studies

The Office of the Dean runs the Faculty of Medicine. The Office of the Dean and Office of the Dean of Studies’ tasks include ensuring the completeness of the teaching on offer, adherence to teaching obligations as well as study and examination organization, creating drafts of study and examination regulations and carrying out evaluations. In the MSCM, as a higher-level of organization the Office of the Dean plays an important role in the implementation of the curriculum, in innovations and in the motivation of lecturers and students. 

##### 6.1.2. Teaching Coordination Group

In order to ensure quality control and further development of the MSCM, a weekly meeting of the professorial MSCM coordination group was initiated. The so-called coordination group, together with the employees of the Office of the Dean of Studies and student representatives, deals with all immediate problems of study and teaching. Discussion topics include, for example, questions and further developments of the curriculum, event evaluation, failure rates in exams, special cases as well as detailed questions that arise in the organization of studies and teaching. 

##### 6.1.3. Cohort coordinators

Each academic year is supervised by a cohort coordinator. The tasks of the cohort coordinators include counseling and supervision of students, timetable design and organization of the academic year in cooperation with the lecturers and module administrators. The introduction of cohort coordinators has introduced a new role to the Office of the Dean of Studies. In addition, there are exam, elective and international coordinators who support counseling in their respective areas [21], [22], [23].

##### 6.1.4. Module leaders

Various institutes and hospitals are involved in the theoretical clinical modules. Their management plays an important role due to the resulting high need for coordination of the individual courses within a block. They are members of the teaching staff of the key hospital/institute involved in the respective course. Together with the lecturers and the cohort coordinators, they design the event contents, also with regard to redundancies and links with other teaching events and compile the exam questions. 

##### 6.1.5. Tutors

The introduction of the Aachen Model Study Course in Medicine required a high degree of willingness on behalf of the lecturers for interdisciplinary work and cooperation amongst themselves and with the Office of the Dean of Studies. In order to avoid redundancies and gaps in the design of courses, intensive and continuous communication is necessary between all participants. The high degree of structuring of medical studies and the associated need for coordination of the teaching content led and still leads to discussions in the continuing development of the curriculum. 

##### 6.1.6. Students/Student Council

The students were very involved in the design and implementation of the MSCM from the very beginning. They participate in the meetings of the Coordination Group and are represented in all committees involved in teaching (for example, the Advisory Council) [24].

## 7. Discussion

The Aachen Model Study Course in Medicine was developed by the Medical Faculty of the RWTH Aachen under strong pressure for innovation and improvement [2]. The complete redesign of the course starting from the first semester onwards posed major challenges for all those involved in the faculty. The implementation of a completely reformed curriculum, such as the model study course, can only succeed through constructive cooperation of the various stakeholders at a faculty. The new focus has been able to address the major flaws of the 2002 Science Council report, student dissatisfaction and the poor performance in the nationwide exam comparison [17].

## 8. Conclusion

The Faculty of Medicine of the RWTH Aachen University will continue the model study course in medicine as long as the formal requirements of the licensing regulations allow this. When the model study course clause expires, elements and experiences of the Model study course may be used for the development of new curricula.

## Competing interests

The authors declare that they have no competing interests. 

## Figures and Tables

**Figure 2:**
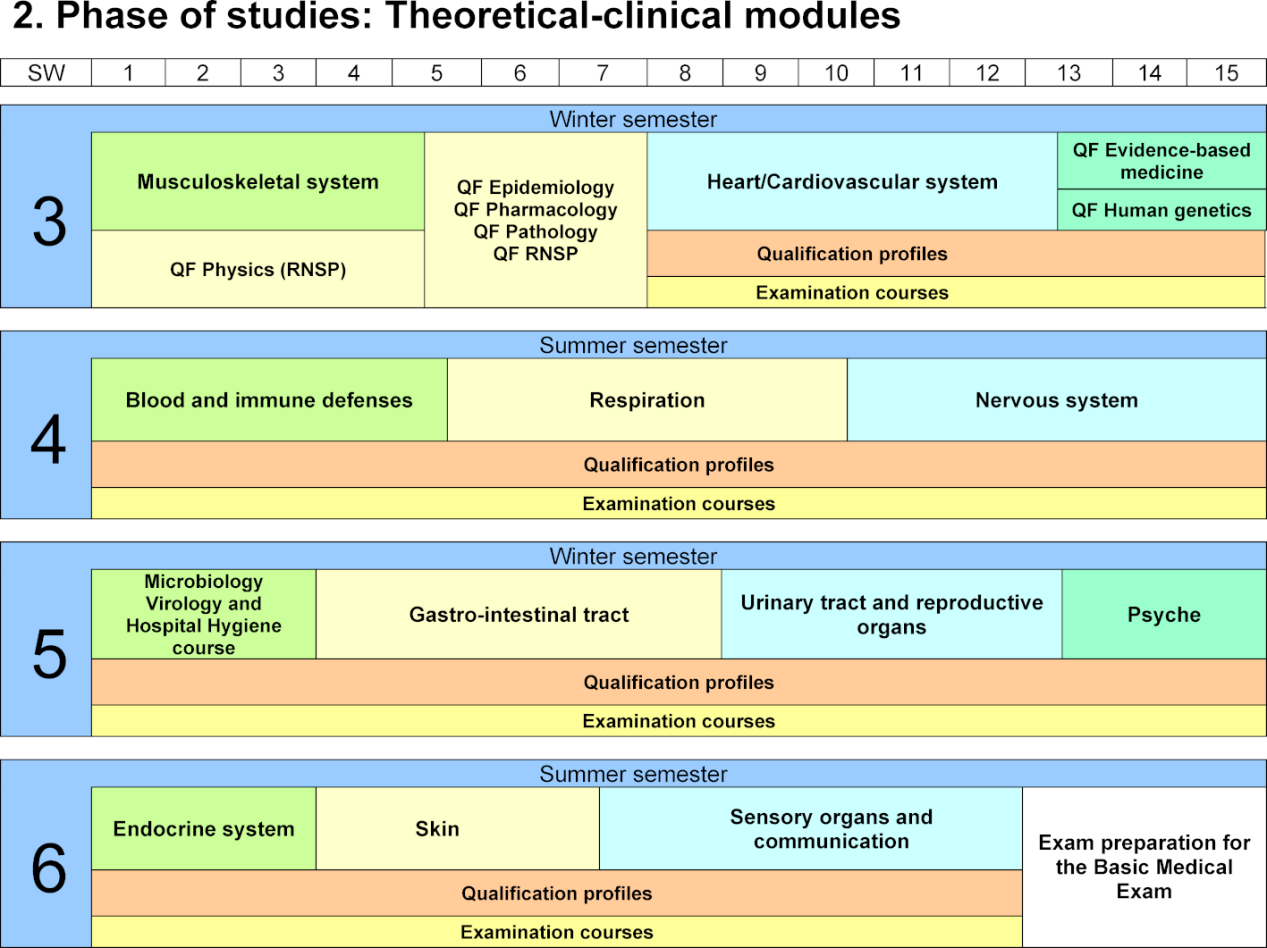
2. Phase of studies: theoretical-clinical modules

## References

[1] Wissenschaftsrat (2014). Stellungnahme zur weiteren Entwicklung der Medizinischen Fakultät der Rheinisch-Westfälischen Technischen Hochschule Aachen.

[2] Groß D, Kleinmanns J, Schwanke E (2016). 50 Jahre Medizinische Fakultät 1966 - 2016, RWTH Aachen.

[3] RWTH Aachen (2018). Studien- und Prüfungsordnung für den Modellstudiengang Medizin der Rheinisch-Westfälischen Technischen Hochschule Aachen mit dem Abschluss "Ärztliche Prüfung" vom 05.11.2008.

[4] RWTH Aachen Profil des Studiums der Humanmedizin an der RWTH Aachen.

[5] Frank JR, Danoff D (2007). The CanMEDS initiative: implementing an outcomes-based framework of physician competencies. Med Teach.

[6] Epstein RM (2007). Assessment in Medical Education. N Engl J Med.

[7] Spreckelsen C, Döpke R, Sárándi I (2014). Zuordnung von Nationalem Kompetenzbasiertem Lernzielkatalog und fakultätsspezifischen Lernzielkatalogen durch automatische Textverarbeitung.

[8] Medizinische Fakultät Mannheim der Universität Heidelberg (2015). Manual für PJ-Betreuer: Informationen rund ums Praktische Jahr.

[9] Stanford University (Speak). Problem-Based Learning.

[10] Davis MH, Harden RM (2003). Planning and implementing an undergraduate medical curriculum: the lessons learned. Med Teach.

[11] Harden RM, Grant J, Buckley G, Hart IR (1999). BEME Guide No. 1: Best Evidence Medical Education. Med Teach.

[12] Gesellschaft für Medizinische Ausbildung, GMA-Ausschuss Prüfungen, Kompetenzzentrum Prüfungen Baden-Württemberg, Fischer MR (2008). Leitlinie für Fakultäts-interne Leistungsnachweise während des Medizinstudiums: Ein Positionspapier des GMA-Ausschusses Prüfungen und des Kompetenzzentrums Prüfungen Baden-Württemberg. GMS Z Med Ausbild.

[13] Savery JR (2006). Overview of Problem-based Learning: De?nitions and Distinctions. Interdiscipl J Probl Based Learn.

[14] Brahm T, Jenert T, Meier C (2010). Hochschulentwicklung als Gestaltung von Lehr- und Lernkultur: Eine institutionsweite Herangehensweise an lehrbezogene Veränderungsprojekte an Hochschulen.

[15] Coombes L, Ricketts C, Freeman A, Stratford J (2010). Beyond assessment: feedback for individuals and institutions based on the progress test. Med Teach.

[16] Nouns ZM, Brauns K, Dany S, Szczyrba B, Wildt J (2008). Progress-Testing - ein Verfahren zur detaillierten Leistungsdarstellung und Lehrevaluation auf Basis der Wissensentwicklung von Studierenden. Prüfungen auf die Agenda!: Hochschuldidaktische Perspektiven auf Reformen im Prüfungswesen.

[17] IMPP Archiv Ergebnisse Medizin.

[18] Bergermann J (2018). Zusammenhänge zwischen der Wahrnehmung spezifischer Strukturmerkmale des Aachener Modellstudiengangs Medizin (AMM) und der Identifikation der Studierenden mit dem Studiengang.

[19] von Jagow G, Lohölter R (2006). Die neue Ärztliche Approbationsordnung: Schwerpunkte der Reform und erste Erfahrungen mit der Umsetzung. Bundesgesundheitsbl.

[20] Bundesministerium für Bildung und Forschung (2017). Masterplan Medizinstudium 2020.

[21] Salden P, Barnat M, Hofhues S, Kenneweg AC, Merkt M, Salden P, Urban D (2013). Der Third Space als Handlungsfeld in Hochschulen: Konzept und Perspektive. Junge Hochschul- und Mediendidaktik: Forschung und Praxis im Dialog.

[22] Carstensen D (2015). Third Space in Hochschulen: Ein Raum für neue Aufgaben. Wissenschaftsmanag.

[23] Meinel FG, Dimitriadis K, Borch Pvd, Störmann S, Niedermaier S, Fischer MR (2011). More mentoring needed?. A cross-sectional study of mentoring programs for medical students in Germany. BMC Med Educ.

[24] Gale R, Grant J (1997). AMEE Medical Education Guide No. 10: Managing change in a medical context: Guidelines for action. Med Teach.

[25] Schwartz P (2001). Problem-based Learning.

